# Clinical, epidemiological characteristics and associated factors of hair greying in Lagos, Nigeria

**DOI:** 10.4314/gmj.v56i1.1

**Published:** 2022-03

**Authors:** Ehiaghe L Anaba, Olusola Ayanlowo, Olufolakemi M Cole-Adeife, Erere Otrofanowei, Ayesha O Akinkugbe, Itohan R Oaku, Ireneh Akwara

**Affiliations:** 1 Department of Medicine, Lagos State University College of Medicine/ Lagos State University Teaching Hospital, Lagos, Nigeria; 2 Department of Medicine, College of Medicine, University of Lagos/Lagos University Teaching Hospital, Lagos, Nigeria; 3 Department of Medicine, Lagos State University Teaching Hospital, Lagos, Nigeria; 4 Department of Medicine, General Hospital, Lagos, Nigeria

**Keywords:** Hair greying, premature hair greying, epidemiological, clinical, Nigeria

## Abstract

**Objective:**

To document the epidemiological, clinical characteristics, believed triggers and associated behaviour in hair greying.

**Design:**

A community based cross-sectional descriptive study was conducted in February 2020 following ethical approval and written informed consent from participants. All participants were clinically evaluated for hair greying, its pattern and location on the scalp. Socio-demographic data were documented. Data was entered and analyzed using the IBM statistics software version 22. Numerical and categorical variables are presented.

**Setting:**

The study was conducted at an urban market in Lagos, Nigeria.

**Participants:**

The study participants comprised 307 adult traders.

**Results:**

The mean age of the 307 participants studied was 42.7±12.8 years. The prevalence of hair greying was 47.6% (51% in males and 45.9% in females). The median (IQR) age of those with grey hair was 52 (44, 59) years. The prevalence of hair greying was 14.8% in those aged 30–34 years and 97.2% in those aged 60 years and above. The prevalence of premature greying was 17.7% and greying before friends and family members was reported at 19.9% and 13%, respectively. Grey hair was diffuse in 81.5%; localized to the frontal area of the scalp in 55.5%. Use of hair dye was noted in 15.8%.

**Conclusion:**

Hair greying is common in the study population. The age at onset is 30 years. Premature hair greying is uncommon in Nigeria. More epidemiological studies of hair greying especially of premature hair greying are needed.

**Funding:**

Funding for this study was provided by the L'Oreal African Hair & Skin Research Grant.

## Introduction

Hair greying (HG), also called canities, is a common phenomenon associated with ageing irrespective of race and gender.^1^,^2^ Epidemiological studies of this common phenomenon are few.^2^ Hair greying if early, can negatively impact the quality of life of some individuals.^3^ There are two forms of HG; premature HG (early-onset) and chronological HG (late-onset).^4^,^5^ The acronym HG is reserved for chronological hair greying. There are documented racial differences in age of onset and age stipulated in the definition of premature HG.^1^,^6^,^7^

Hair greying is said to be premature (PHG) when the age at onset is less than 20 years, 25 years, and 30 years, respectively, in Caucasians, Asians, and Africans.^6^,^7^

Also, Africans are reported to grey later than Caucasians with respect to late-onset hair greying (HG).^1^,^8^ While the average age of onset of HG in Africans is 43.9 years, in Caucasians, it is 34.0 years.^1^ The cause of HG is unknown, but it is attributed to decreased melanogenesis and defective melanosomal transfer in hair follicles.^2^,^9^

The risk factors identified for HG include chronological age, smoking, alcohol, oxidative stress, metabolic diseases, trace elements and stress.^2^,^9^–^12^The few epidemiological studies available reveal that the prevalence of premature hair greying (PHG) varies between 7.5% and 36.4%.^4^,^5^ On the other hand, the prevalence of HG is 69% and 75%.^4^,^13^

The prevalence of HG in females varies from 41.1% to74.8% and in males from 59.9% to 75.6%.^4^,^13^ The reported mean age of those who have HG is 50.8 years.^14^ Prevalence of HG varies with age and is reported to increase with age.^4^,^13^,^14^ The reported prevalence based on age is as follows; 1.6% in those aged 10–19 years, 7.4% in those aged 20–29years, 14.8% in those aged 30–39 years, 19.2% in those aged 40–49 years, 28.1% in those aged 50–59 years and 28.8% in those aged 60 years and over.^14^ The mean age of onset of HG is 33 years, lower in females (31 years) than in males (33 years).^4^ Panhard et al in their worldwide survey of HG concluded that, at age 50 years, 6–23% of individuals would have HG.^8^ Clinically, the most common anatomical location of HG is the temporal region in 83.7%, followed by the parietal (64.2%), frontal (60.8%) and occipital (58.4%) in descending order.^4^ Hair dyeing to camouflage HG is common in individuals with HG, especially if it is PHG.14 Jo et al. In their study of 620 Koreans who had HG reported a 43.9% prevalence of hair dyeing.^14^

Although HG is a common phenomenon, there is a dearth of literature regarding HG in Nigeria. Its prevalence and the beliefs and behavioural practices of individuals with HG are unknown. We, therefore, set out to determine the prevalence of HG and of PHG, age at onset of HG, the believed triggers of HG and the associated behaviours.

## Methods

This cross-sectional descriptive study of 307 adult traders following ethical approval by the Lagos State University Teaching Hospital (LREC/06/10/1297) was conducted in February 2020. Written informed consent was obtained from all the participants. The study was conducted amongst traders at a designated market that had a total of 305 stalls. A pre-tested self-administered questionnaire was given to all participants. The questionnaire had questions on age, gender, presence of grey hair, age at onset of grey hair, the onset of grey hair before age-matched friends and family members, excessive grey hair, use of hair dye and what the participants perceive to be trigger factors for greying.

The convenient sampling method was deployed in this study. All the traders in the market were serially numbered and inspected for grey hair and, the location of the grey hair. The diagnosis of grey hair was strictly by observation. For this study, any participant who had more than one strand of grey hair was recruited into the study. Grey hair was noted as;

- Localized if there were just a few strands localized to any area of the scalp (temples, frontal, vertex, occiput)
- Diffuse if strands were all over the scalp and could not be seen as being restricted to a part of the scalp.

Premature hair greying was noted as anyone with the on-set of HG at age less than 30 years.^7^

Any individual who had a pigmentary disorder or had a scalp disorder that would influence the hair colour was excluded from the study. Data was entered and analyzed using the IBM statistics software version 22. Numerical and categorical variables are presented as means and percentages respectively.

## Results

Three hundred and seven participants with a mean age of 42.7±12.8 years were examined and 146 of them had grey hair giving a prevalence of 47.6% (146/307). Specifically, the prevalence of grey hair was 51% in males (51/100) and 45.9% in females (95/207). Grey hair was not found in participants less than 29 years and after age 60 years, all the participants had grey hair. Table 1

**Table 1:** Age and gender distribution of participants with and without grey hair

Variable	Grey hair n=146 (%)	No Grey hair n=161 (%)	Total n=307 (%)
**Age group** **(years)**			
< 29	0 (0.0)	51 (31.7)	51 (16.6)
30 – 39	15(10.2)	62(38.5)	77 (25.1)
40 – 49	45(30.8)	39 (24.3)	84 (27.4)
50 – 59	51(34.9)	9 (5.5)	60 (19.5)
≥60	35(24.0)	0 (0.0)	35 (11.4)
**Mean ±SD**	51.5±10.1	34.3±8.9	42.7±12.8
Range	32 –75	19 – 55	19 –78
**Gender**			
Male	51 (34.9)	49 (30.4)	100 (32.6)
Female	95 (65.1)	112 (69.6)	207 (67.4)

The Median (IQR) age of those who had grey hair was 52 (44, 59) years (range of 35–75 years), 51 (44, 57) years in males and 52 (43, 60) years in females. The prevalence of grey increased with age, 14.8% in 30–34-year olds and 97.2% in 60-year olds and above. Amongst those who had grey, 65.1% were females. The prevalence of premature hair greying (PHG) was 17.7%. The mean age at onset of grey hair was 39.5±14.5 years. The age at onset of grey hair was < 30 years in 17.7% and 6.3% at 60 years and above (Tables 2).

**Table 2:** Age, gender, and age-specific prevalence of participants with grey hair

Variable	Male n=51(%)	Female n=95(%)	Total n=146 (100%)
**Age group** **(years)** 30 – 39 40 – 49 50 – 59 ≥ 60 Median (IQR)	4 (7.8) 17(33.3) 21(41.2) 9 (17.6) 51(44,57)	11(11.6) 28(29.5) 30(31.6) 26(27.4) 52(43,60)	15 (10.3) 45 (30.8) 51 (34.9) 35 (23.9) 52 (44, 59)
**Age at onset **of grey hair** < 30 30 – 39 40 – 49 50 – 59 ≥ 60 Median (IQR)	6 (17.2) 8 (22.9) 12(34.3) 8 (22.9) 1 (2.9) 40(31,50)	11(18.1) 14(23.0) 13(21.3) 18(29.5) 5 (8.2) 42(34,50	17 (17.7) 22 (22.9) 25 (26.0) 26 (27.1) 6 (6.3) 41(32,50
**Age specific** **prevalence** <30 30 – 34 35 – 39 40 – 44 45 – 49 50 – 54 55 – 59 ≥60	0/51 (0.0) 4/27(14.8) 11/50(22.0) 25/47(53.2) 20/37(54.1) 31/38(81.6) 20/21(95.3) 35/36(97.2)		

**Not known in 35 participants

One hundred and forty-six participants were clinically evaluated to have hair greying. However, only 131 (89.7%) participants gave a history of hair greying, and only these 131 participants answered the follow-up questions. Most of the participants (82.2%) did not know what triggers hair greying.

Clinically, grey hair was diffuse in 81.5%. In those in whom it was localized, it was localized to the frontal area of the scalp in 55.5%. Use of hair dye was noted in 15.8%. Greying before friends were reported in 19.9% and hair dye use in 15.8% (Table 3)

**Table 3:** History and clinical characteristics of grey hair

Variable	Frequency (n=146)	Per cent (%)
**Greying earlier than friends**		
Yes	29	19.9
No	73	50.0
I don't know	44	30.1

**Greying started before family** **members**		
Yes	19	13.0
No	87	59.6
Don't know	40	27.4

**Have fast greying**		
Yes	30	20.5
No	80	54.8
Don't know	36	24.7

**Triggering factors of excessive** **grey hair**		
Emotional stress	13	8.9
Excessive sun exposure	1	0.7
Hard work	10	6.8
Chronic medical condition	2	1.4
Don't know	120	82.2

**Use of hair dye**		
Yes	23	15.8
No	123	84.3

**Pattern of grey hair**		
Diffused	119	81.5
Localized	27	18.5

**If localized (where)**	**n=27**	
Temples	6	22.2
Frontal	15	55.5
Vertex	8	29.6
Occiput	2	7.4

## Discussion

Hair greying is a common phenomenon associated with ageing irrespective of race and gender with a worldwide occurrence.^1^,^2^ In this study, the perceived triggers for hair greying and the associated clinical and epidemiological characteristics are demonstrated.

Hair greying was prevalent in almost half of the population studied and the prevalence was higher in males. The prevalence of HG in this study is lower than that reported in two other population studies.^4^,^13^ A couple of reasons may have accounted for this difference in prevalence.

These two studies were conducted in Asians and Caucasians who are reported to have an earlier age of onset of HG than Africans.^7^ Also, these two studies had individuals who had grey hair at 20 years and below as against no observed grey hair in individuals less than 30 years of age.

The prevalence of HG was found to increase with age in consonance with other studies of HG prevalence.^4^,^13^,^14^ This study outcome confirms the increase in chronological age as one of the factors responsible for HG.^9^

The prevalence of HG was higher in males than females, similar to the study reports from other epidemiological studies.^4^,^8^,^13^ These studies were conducted in participants of different races and had the same gender outcome. Further studies to ascertain the reasons for a predominant male HG may be required. The median age of those with HG was the same in both genders. This median is lower than that reported by Jo et al.^14^ Jo et al However, our study did not find any gender difference in age in their study.^14^

The prevalence of PHG was low in this study compared to that reported in other studies and nobody below age 29 years had HG.^5^ In keeping with this low prevalence of PHG, the reported history of greying before family members and friends was low. This is unlike the studies from Turkey and Maryborough in which individuals younger than 29 years had grey hair.^4^,^13^

The difference in the study report can be attributed to the racial difference in age at the onset of HG.^1^,^6^,^7^ The mean age at onset of HG was 39 years, similar in males and females. This is higher than the 33 and 34 years reported by other authors.^1^,^4^ In addition, Acer et al reported a delay in age at the onset by 2 years in females.^4^

Knowledge on triggers for HG was poor as this was not known in the majority of the participants. Emotional and physical stress (working too hard) was the most indicated triggers. Stress has been reported as a risk factor for greying, especially in PHG.^2^,^11^ The practice of hair dye was low compared to the study by Jo et al in Korea.^14^ The authors are unable to account for this difference in behaviour, but we opine that Koreans being more cosmetically aware and inclined than Nigerians may be the reason. There are no African nor Nigerian studies to compare this study outcome with.

The observed pattern of HG was diffuse in most individuals. In the few with increased localization to a scalp area, it was mostly to the frontal scalp. Hair greying is diffuse in most epidemiological studies, just like this study.^4^,^13^,^14^ In the only study on location on the scalp, the temporal region was more affected.^4^ This study by Acer et al did not specifically look at increased localization of HG.^4^ Limitations to this study include the few participants available for study and the scarce literature on epidemiological studies of HG in this clime.

## Conclusion

The prevalence of hair greying is high, and the age at on-set is 30 years. Premature hair greying is uncommon in Nigeria. The pattern of hair greying is mostly diffuse, and when localized, it is more common in the frontal region of the scalp. More epidemiological studies of hair greying, especially premature hair greying, are needed.
